# Implicit and Explicit Evaluation of Visual Symmetry as a Function of Art Expertise

**DOI:** 10.1177/2041669518761464

**Published:** 2018-03-08

**Authors:** Hanna Weichselbaum, Helmut Leder, Ulrich Ansorge

**Affiliations:** Faculty of Psychology, University of Vienna, Austria

**Keywords:** symmetry, explicit rating scale, Implicit Association Test, art history, art experts

## Abstract

In perception, humans typically prefer symmetrical over asymmetrical patterns. Yet, little is known about differences in symmetry preferences depending on individuals’ different past histories of actively reflecting upon pictures and patterns. To address this question, we tested the generality of the symmetry preference for different levels of individual art expertise. The preference for symmetrical versus asymmetrical abstract patterns was measured implicitly, by an Implicit Association Test (IAT), and explicitly, by a rating scale asking participants to evaluate pattern beauty. Participants were art history and psychology students. Art expertise was measured using a questionnaire. In the IAT, art expertise did not alter the preference for symmetrical over asymmetrical patterns. In contrast, the explicit rating scale showed that with higher art expertise, the ratings for the beauty of asymmetrical patterns significantly increased, but, again, participants preferred symmetrical over asymmetrical patterns. The results are discussed in light of different theories on the origins of symmetry preference. Evolutionary adaptation might play a role in symmetry preferences for art experts similarly to nonexperts, but experts tend to emphasize the beauty of asymmetrical depictions, eventually considering different criteria, when asked explicitly to indicate their preferences.

Visual symmetry has long been known to be preferred over asymmetry (e.g., Hubbell, 1940; Pecchinenda, Bertamini, Makin, & Ruta, 2014; Treder, 2010). By means of the following study, we tested the generality of this preference. Based on theoretical considerations on art history (cf. Darvas, 2003; McManus, 2005), we examined the proposition that asymmetry has a better standing with art history experts than art nonexperts.

For a long time, beauty has been believed to be connected to symmetry. For example, the philosopher and mathematician Hermann Weyl (1952) concluded, “Beauty is bound up with symmetry” (p. 3). Weyl’s statement concerned bilateral symmetry, which is the reflection of one half of an image on an imagined straight line (cf. Darvas, 2003). This is also the most frequently investigated form of symmetry (Treder, 2010). Indeed, in line with theoretical speculations, empirical research has shown that symmetry plays a role for the aesthetic experience of an object or image (Leder, Belke, Oeberst, & Augustin, 2004). It is the best predictor of preferences for abstract patterns (Jacobsen & Höfel, 2002), and even a small deviation in symmetry diminishes the appreciation of a visual pattern (Gartus & Leder, 2013). Symmetry preferences are not only found for patterns. They extend to the evaluation of human faces (Cárdenas & Harris, 2006; Halberstadt & Rhodes, 2003) and dynamic stimuli (Wright & Bertamini, 2015) and can be found in animals as well (e.g., Swaddle & Cuthill, 1994). Because of its ubiquity, symmetry preference is usually attributed to evolutionary adaptation (cf. Cárdenas & Harris, 2006). For instance, face or body symmetry, as indicating good genes, might be an indicator of the quality of a possible mating partner (Møller, 1992). Another account is the fluency hypothesis by Reber, Schwarz, and Winkielman (2004), stating that symmetry is preferred because it can be processed easier and more fluently, comprising both speed and accuracy (Reber, Wurtz, & Zimmermann, 2004), compared with the processing of asymmetry.

In the present study, we wanted to test the generality of the symmetry preference. We did not doubt that symmetry is ubiquitous and prominent in everyday design (Lidwell, Holden, & Butler, 2010; McManus, 2005) as well as in the art and design of different cultures and historical epochs (Washburn & Crowe, 1988). However, we hypothesized that experience and knowledge in reflecting upon visual patterns and images, such as in art expertise, might alter the preference for symmetry (cf. Lindell & Mueller, 2011; Silvia, 2006). Our hypothesis is, on the one hand, based on theoretical considerations on aesthetic experience and, on the other hand, backed up by empirical findings: take for instance the information-processing model of the aesthetic experience by Leder et al. (2004). In their model, the authors propose that aesthetic experience of an art work or any other aesthetically relevant object comprises automatic processes (for instance, perceptual analyses of the object) followed by a more deliberate cognitive mastering evaluation. This latter process describes the retrieval of knowledge concerned with the object at hand. Both the automatic and the deliberate processes contribute to aesthetic judgments and emotions. Therefore, according to Leder et al. (2004), art experts should show early fast, automatic preferences similarly to nonexperts but might use different art-evaluation criteria that come into play during later, more deliberate processing stages only, when asked to give explicit aesthetic judgments. Indeed analogous stage-dependent differences have been demonstrated in other art-related domains: Experts and nonexperts show similar emotional responses to art of negative content but differ in terms of their explicit evaluations of such artworks (Leder, Gerger, Brieber, & Schwarz, 2014). Likewise, Lindell and Mueller (2011) suggested that with art training, the importance of symmetry for aesthetic evaluation could decrease, and McManus (2005) outlined the importance of asymmetry in visual art, additionally stating that art historians would agree “that although symmetry is indeed attractive, there is also a somewhat sterile rigidity about it, which can make it less attractive than the more dynamic, less predictable beauty associated with asymmetry” (p. 157). In line with these theoretical considerations, in a study by Barron (1952; as cited in McWhinnie, 1968), artists preferred asymmetrical-complex patterns over symmetrical-simple ones. This latter finding, however, could also be due to influences of complexity as art training also alters the influence of complexity on aesthetic evaluations (Silvia, 2006).

In the current study, we therefore tested whether automatic and more explicit symmetry preferences vary as a function of art expertise. Based on theoretical considerations of Leder et al.’s (2004) model and the reviewed findings, we hypothesized that both art experts and nonexperts could show a preference for symmetrical over asymmetrical patterns when measuring the more automatic processes. However, when participants deliberately reflect upon a pattern’s beauty, art experts could show a higher preference for asymmetrical patterns compared with nonexperts due to the importance of asymmetrical depictions in art as outlined earlier (cf. Leder et al., 2004; McManus, 2005).

To test the generality of the preference for symmetry, in the present study, we compared the preferences by implicit and explicit measures (cf. Makin, Pecchinenda, & Bertamini, 2012). We reasoned that implicit measures reflected the fast, more automatically generated symmetry preference shared by art experts and nonexperts, and that the explicit measure tapped into the more time-consuming, deliberate preferences which could differ depending on art expertise. To that end, we tested participants of different levels of art expertise, with level assignment based on an art-expertise questionnaire. Participants were students of two different faculties, psychology and art history, so as to create sufficient variability in art expertise within our sample. Explicitly, symmetry preference was measured by using a rating scale for symmetrical and asymmetrical abstract patterns. Implicitly, symmetry preference was (indirectly) measured by using a variant of the Implicit Association Test (IAT; Greenwald, McGhee, & Schwartz, 1998).

The IAT uses words of positive or negative valence and targets for which the valence is to be tested—in our case, symmetrical and asymmetrical abstract patterns. In our study, participants had two tasks to discriminate between the positive versus negative valence of words on the one hand and between symmetrical and asymmetrical target patterns on the other. In both tasks, the same two alternative key presses were used for the discriminations. In the *compatible block*, positive words and symmetrical patterns (and, likewise, negative words and asymmetrical patterns) required the same key press. In contrast, in the *incompatible block*, positive words and asymmetrical patterns (and negative words and symmetrical patterns) required the same key press. If participants relate symmetry to positive valence (positive words), we expected shorter correct response times (RTs) in the compatible compared with the incompatible block because the more-related concepts would require the same response and the less-related concepts would require different responses in compatible but not in incompatible blocks. This should facilitate retrieving and subsequently choosing the correct response in compatible blocks as compared with incompatible blocks. The corresponding RT difference is known as the *IAT effect* (e.g., Makin et al., 2012). Note that the IAT effect does not directly reveal implicit preferences but shows how strongly two concepts are related. In our study, the IAT effect should reveal the relatedness of symmetrical patterns and words with positive valence (as well as asymmetrical patterns and words with negative valence) and might therefore serve as a direct measure of valence and as an indirect measure of preference of symmetrical over asymmetrical patterns.

Based on existing research, art experts might show a different preference compared with nonexperts (cf. Lindell & Mueller, 2011; Silvia, 2006). If art expertise indeed changes the way symmetry is evaluated, art experts might either evaluate symmetrical and asymmetrical patterns more similarly or even show a preference for asymmetrical over symmetrical patterns. However, this finding could be restricted to explicit preference measures and in an implicit measure, art experts might show symmetry preferences as everyone else because these automatically generated, fast symmetry preferences could reflect preferences of evolutionary adaptation. If that is the case, the comparison of implicit and explicit preferences allows testing whether art expertise comes with a preference toward asymmetry in explicit measures only. This could be based on a shift of criteria, for instance, because only experts but not nonexperts would be able to incorporate into their explicit aesthetic appreciations their many positive experiences with asymmetrical images and objects for revisions of their initial implicit symmetry preferences. However, art experts might also even explicitly discard “*simple*” principles of beauty for their explicit appreciations.

Yet, before the IAT measures can be interpreted correctly, a potential method-specific complication of the IAT needs to be noted. According to the rationale of the IAT, shorter correct RTs are to be expected in the compatible compared with the incompatible condition because of an easier response selection in the compatible than incompatible blocks (cf. Makin et al., 2012). However, Mierke and Klauer (2001, 2003) pointed out that this RT difference between compatible and incompatible blocks could also be due to a more deliberate process—the recoding of the tasks into one joint task representation in the compatible block only, so that trial-to-trial task switching costs would only be present in the incompatible block. According to this explanation, in the compatible block, the same responses to positive words and targets (in our case to symmetrical patterns) and the same alternative responses to negative words and targets (in our case to asymmetrical patterns) would invite participants to judge all stimuli by their valence, so as to allow recoding of the two tasks into one joint, single task representation. However, in the incompatible block, the key presses for positive words and symmetrical patterns (and for negative words and asymmetrical patterns, respectively) are different. Therefore, in the incompatible block, participants would have to keep two separate task representations, with the consequence that when the stimulus switches from one trial to the next, the task would switch, too. As trial-to-trial task switches create a switching cost in the RTs of the second trial of the two trials (Allport, Styles, & Hsieh, 1994; Jersild, 1927; Rogers & Monsell, 1995), the IAT effect could also be due to the selective task switching costs in incompatible trials.

Task coding differences, however, could tap into more explicit and deliberate recoding of the tasks and, hence, might not allow testing the expected differences between implicit and explicit measures. Therefore, we made sure that switching costs could be found in compatible blocks, too. We also tested if IAT effects were present in target-repetition conditions of compatible *and* incompatible blocks. In addition, switching costs could show stable interindividual differences (Kray & Lindenberger, 2000; Mierke & Klauer, 2003) that might have little to do with symmetry preferences and with art expertise. This was an additional reason that we wanted to check for this method-specific complication. We did so by the randomized presentation of to-be-evaluated words and to-be-discriminated pattern targets within blocks. Doing so allowed us to analyze task (or target type) repetition trials, where on two successive trials, the type of the stimulus (word vs. pattern) and thus the task was the same (e.g., two pattern trials in succession) and to compare them to task switching trials, where the type of stimulus and, hence, the task changed (e.g., one word trial followed by a pattern trial).

Note that the task switching account by Mierke and Klauer (2001, 2003) does not call into question that the IAT reveals preferences, as the compatible block invites judgments solely based on valence only if participants evaluated the targets (here: the symmetrical patterns) positively in the first place. However, the origin of this preference could then be rather an explicit and deliberate process. Thus, to find switching costs regardless of compatibility and to demonstrate symmetry preferences regardless of whether the task repeats or not would support our conclusion that these preferences would not be due to explicit and deliberate recoding in advance of the stimuli only.

## Method

### Participants

In total, 79 participants took part. Participants were 49 students of psychology (*M*_age_* = *20.37 years, age: 18–28 years) and 30 students of art history (*M*_age_* = *25.33 years, age: 22–44 years) at the University of Vienna. They participated voluntarily in exchange for course credits or a payment. All had normal or corrected-to-normal vision and no dyschromatopsia as assessed by Ishihara color plates. Participants filled out and signed an informed consent prior to the experiment. They were informed that they could withdraw at any time during the experiment without further consequences, that participant and data collection were fully anonymous, and that the data were to be used for a scientific publication (unless they objected to this until publication). We were cautiously monitoring the well-being of our participants but did not observe any inconvenience. For each participant, the whole study lasted about 30 min. As the study was not in any way harmful, medically invasive, and did not induce stress among participants, no ethical approval was sought, which is also in accordance with University of Vienna’s requirements regarding ethical approval.

### Apparatus and Software

The experiment took place at the Faculty of Psychology of the University of Vienna. All three parts of the experiment (the expertise questionnaire, the explicit rating scale, and the IAT) were administered on a 19-in. TFT monitor with a resolution of 1280 × 1024 pixels and a refresh rate of 60 Hz. A keyboard and a standard USB computer mouse were placed in front of the participants. A table lamp served as an indirect light source behind the monitor. A chin rest and forehead strip ensured a viewing distance of 57 cm. Stimuli presentation was programed using the SR Research Experiment Builder (version 1.10.165; SR Research, 2004). Data were analyzed using the R programming software (version 3.2.4 Revised, R Core Team, 2016), with the following additional packages: the ‘*MASS*’ package (Venables & Ripley, 2002) for defining contrasts, the ‘*lmerTest*’ package (Kuznetsova, Brockhoff, & Christensen, 2016) for running linear mixed models, the ‘*effects*’ package (Fox, 2003) for plotting the results, the ‘*lsmeans*’ package (Lenth, 2016) for comparing slopes of fitted lines, and the ‘*schoRsch*’ package (Pfister & Janczyk, 2015) for removing RT outliers.

### Stimuli

**Figure 1.**
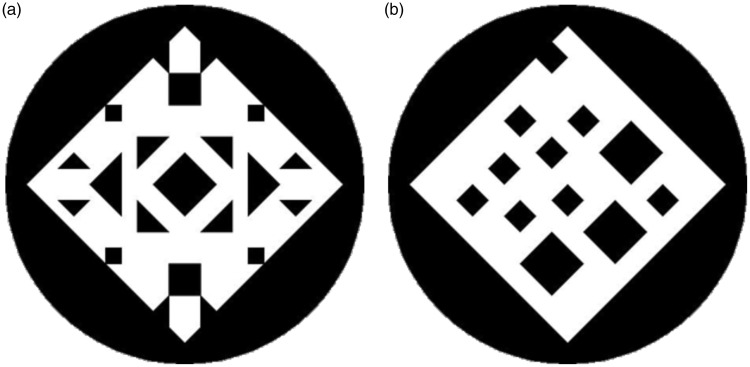
Example stimuli used in our study originally produced by Jacobsen and Höfel (2001, 2002). (a) On the left, you can see a symmetrical pattern and (b) on the right, an asymmetrical one.

The symmetrical and asymmetrical patterns were originally produced by Jacobsen and Höfel (2001, 2002; for example stimuli, see Figure 1). These stimuli were chosen because of their sensitivity with regard to aesthetic preferences (Jacobsen & Höfel, 2001; Tinio & Leder, 2009). The stimuli partly differ from one another in the number of symmetry axes depicted, the numbers of elements contained within the pattern, and so on. However, because we used general linear mixed models (GLMM) to analyze the results, the stimulus-specific variance was explicitly considered. In detail, each such stimulus consists of small black graphic elements on a white background square positioned on a black disc (8.8 cm in diameter). Out of their 252 stimuli, we chose 80 complex patterns (i.e., patterns with more than 10 individual elements) used in Tinio and Leder (2009). As in Tinio and Leder, stimuli were categorized as symmetrical, when symmetry was present along at least one axis (bilateral symmetry). Otherwise they were categorized as asymmetrical.

For the IAT, we used 10 positive and 10 negative words from the Berlin Affective Word List-Reloaded by Võ et al. (2009; for the list of the 20 words and their translations, see Table 1). We chose only nouns with a clear positive or negative valence and without German umlaut (“ä,” “ö,” “ü”) or “ß.” Valences in that study were originally rated from −3 to +3 (Võ et al., 2009), we excluded words with a weaker valence, between −1.99 and +1.99. Subsequently, we chose words with approximately the same number of letters and syllables. The remaining words had five or six letters and two syllables. Words that differed significantly from the others in their frequency of usage and imageability were excluded. The remaining 10 positive and 10 negative words did not differ significantly in their arousal values. All stimuli (patterns and words) were presented on a gray background. All 20 words used in the IAT as well as the instructions were written in bold Courier New font of Size 20.

**Table 1.** List of German Words With Positive and Negative Valence and Their English Translations.

Words with positive valence		Words with negative valence	
German	English translation	German	English translation
RETTER	savior	TYRANN	tyrant
JUBEL	cheer	STRAFE	penalty
TALENT	talent	GREUEL	atrocity
FEIER	celebration	TRAUER	grief
PARTY	party	OPFER	victim
GEWINN	profit	ABGAS	exhaust gas
HUMOR	humor	BETRUG	fraud
HOBBY	hobby	PLAGE	plague
REISE	journey	ARREST	arrest
FREUDE	joy	BEFEHL	command

*Note.* The German words were selected from the BAWL-R by Võ et al. (2009; Võ, Jacobs, & Conrad, 2006). Translation into English by the first author.

### Procedure

The experiment consisted of three parts: an art-expertise questionnaire to classify participants according to their art expertise, an explicit rating scale, and the IAT for the measurement of implicit preferences. The order of the three parts was counterbalanced based on a recommendation by Nosek, Greenwald, and Banaji (2007).

#### Expertise questionnaire

Participants filled out the computer version of an art-expertise questionnaire created at the Vienna lab. The questionnaire consisted of general questions concerning art interest, two parts on knowledge about art, and a last part with demographic questions. For our study, we analyzed the parts on art knowledge. The first part consisted of 10 multiple choice questions with one out of four possible answers correct. The second part consisted of eight works of art, and participants had to indicate whether they knew the art work and to name the artist and the art style (see Appendix A for these two parts of the art-expertise questionnaire). The sum of correct answers of the two parts (including answering the questions on knowing an art work) represented the individual result on the art-expertise questionnaire. As the first part consisted of 10 questions, the second part of eight art works with three questions each, and as each correctly answered question counted as one point, the highest possible score was 34.

#### Explicit rating scale

**Figure 2.**
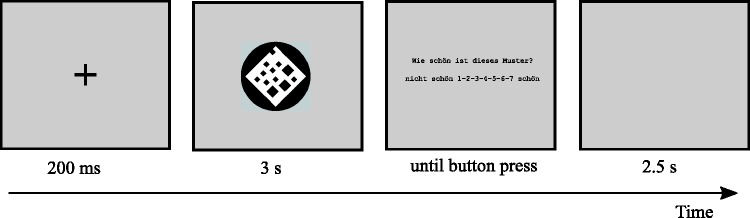
Example trial of the explicit rating part with an asymmetrical pattern. Stimuli are not drawn to scale. On the third display, the German question, “Wie schön ist dieses Muster?” (English translation: “How beautiful is this pattern?”), plus scale, “nicht schön 1-2-3-4-5-6-7 schön” (English translation: “not beautiful 1-2-3-4-5-6-7 beautiful”), was shown.

Participants got the instruction to evaluate each pattern according to its perceived beauty on a rating scale from 1 (*not beautiful*) to 7 (*beautiful*). Subsequently, they were introduced to the rating scale and executed 12 practice trials that were not further analyzed. After practice, there were 68 analyzed trials. Each trial started with the presentation of a black fixation cross at screen center for 200 ms. Next, a pattern was presented at screen center for 3 s, followed by the rating scale. The rating scale was presented until the participant pressed a key from 1 to 7 on the number pad. Before the next trial, a blank screen was presented for 2.5 s. The 80 stimuli used were randomly assigned to the practice and the analyzed trials so that in each part, there was the same amount of symmetrical and asymmetrical patterns. For all participants, we used the same stimuli for the practice trials. Therefore, for all participants, stimuli used in the analyzed trials were also the same (but different from the practice stimuli). An example trial of the explicit rating scale is shown in Figure 2.

#### Implicit Association Test

**Figure 3.**
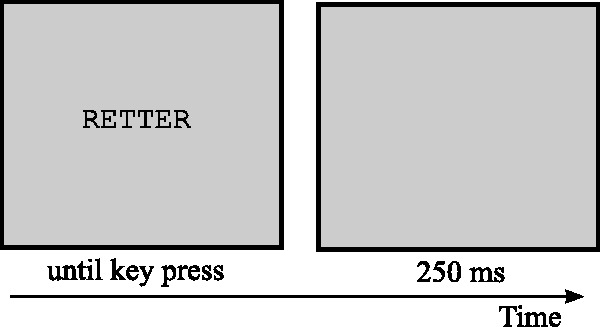
Example of a trial in the IAT showing a positive word. The German word “Retter” stands for “savior.” Stimuli are not drawn to scale.

As we only needed 10 positive and 10 negative words as well as 10 symmetrical and asymmetrical patterns, we randomly picked 10 symmetrical and 10 asymmetrical patterns out of the ones used in the analyzed trials of the explicit rating scale. The patterns used were the same for all participants. Participants got the instruction to press a key (the left or right arrow key) in response to each stimulus’ property, either the valence of a word or the symmetry of a pattern. The mapping of the key presses was counterbalanced across participants. The IAT consisted of two training blocks for patterns and words, respectively, one experimental block, followed again by one training block for patterns, and finally, a second experimental block. Each trial started with the presentation of one (positive or negative) word or (symmetrical or asymmetrical) pattern at screen center. Participants had to press the right or left arrow key in response to the stimulus. After pressing the key, a blank screen was presented for 250 ms before the next trial started if the participant answered correctly. If the participant pressed the wrong key, the German word for *incorrect* (*falsch*) appeared in the middle of the screen for 500 ms, followed by a blank screen for 250 ms before the next trial started. The words to be evaluated according to their valences were presented in capital letters. An example of a trial is shown in Figure 3. In sum, the IAT consisted of 360 trials.

The training blocks for the words as well as for the patterns consisted of 40 trials each—all 20 stimuli (words or patterns) were presented twice, in random order. The order of these two training blocks was counterbalanced across participants.

The training blocks were followed by the first experimental block. This was either the compatible or the incompatible block because the key mapping was the same as in the training blocks. For instance, if, in the training blocks, one participant had to press the same arrow key for symmetrical patterns and positive words and for asymmetrical patterns and negative words, the compatible block followed subsequently (because the compatible block is defined as having the same key press for symmetrical patterns and positive words). In contrast, when asymmetrical patterns and positive words had to be answered with the same key, the first experimental block was the incompatible block. In each experimental block, all 20 words and 20 patterns were presented three times and in random order.

After the first experimental block, a second training block for the patterns was presented. In this training block, participants learned a reversed stimulus-response mapping for the patterns compared with the first training block for patterns. Again, all patterns were presented twice in random order.

After this second training block for patterns, the second experimental block started. Now, the stimulus-response mapping for patterns was similar to the one in the second training block for patterns, but the stimulus-response mapping for words remained the same as in the first training block for words (and as in the first experimental block). Therefore, if the first experimental block was the compatible one, as the stimulus-response mapping for patterns was now reversed, the second experimental block was the incompatible one, and vice versa. Again, all 20 words and 20 patterns were presented three times, and the trials were presented in random order. The structure of the IAT is shown in Table 2.

**Table 2.** Example Procedure of the Implicit Association Test.

Block	Block type	Left key	Right key
1	Training words	Positive word	Negative word
2	Training patterns	Symmetrical pattern	Asymmetrical pattern
3	Compatible block	Positive word or symmetrical pattern	Negative word or asymmetrical pattern
4	Training patterns	Asymmetrical pattern	Symmetrical pattern
5	Incompatible block	Positive word or asymmetrical pattern	Negative word or symmetrical pattern

*Note*. The order of Blocks 1 and 2 as well as the stimulus-response mapping and the order of the compatible and incompatible blocks were counterbalanced across participants. This version is a variant of the original method by Greenwald et al. (1998) and the depiction is inspired by the one used in Makin et al. (2012).

## Results

The data underlying the results of the expertise questionnaire, the explicit rating scale, and the IAT are available at Weichselbaum, Leder, and Ansorge (2017).

### Expertise Questionnaire

**Figure 4.**
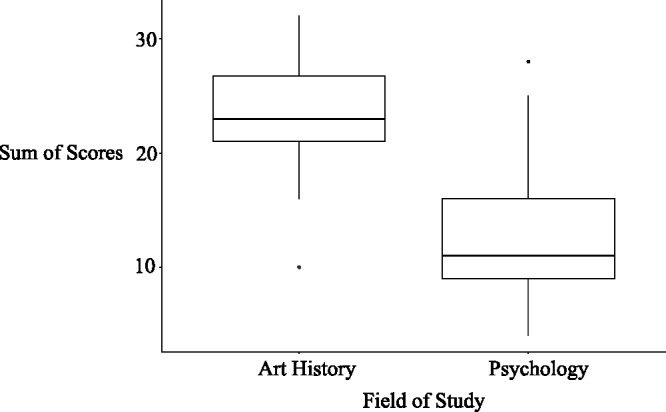
Boxplot showing the sum of scores of the art-expertise questionnaire separated by the field of study.

The highest possible sum of scores on the expertise questionnaire was 34. The mean of all 79 participants was 16.76, the lowest sum of scores was 4 and the highest 32. From lowest to highest scores, art expertise increased. A two-group *t* test showed that art history students had a significantly higher sum of scores (23.43) compared with psychology students (12.67), *t*(66.53) = 9.65, *p* < .001, *d* = 2.21. As shown in Figure 4, the field of study is not a definite indicator of the sum of scores as there are some psychology students having a higher sum of scores compared with some art history students. Therefore, we included the sum of scores, and not the field of study, as a predictor variable in order to adequately account for the influence of participants’ art expertise.

### Explicit Rating Scale

**Figure 5.**
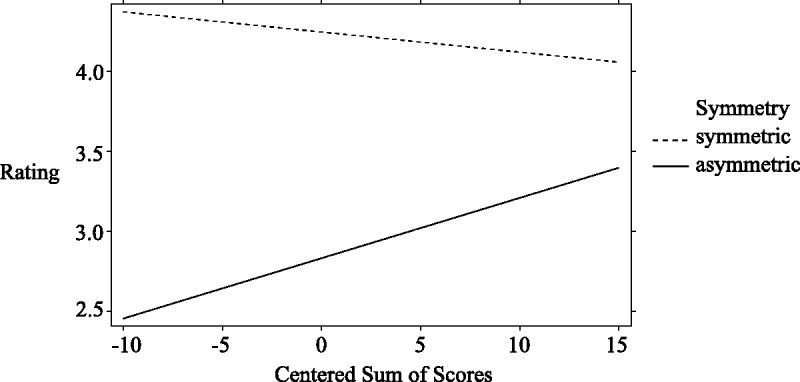
Interaction between the patterns’ symmetry and the centered sum of scores of the art-expertise questionnaire on the explicit rating scale. A higher centered sum of scores signifies higher art expertise. Ratings on the patterns’ perceived beauty were done on a scale from 1 (*not beautiful*) to 7 (*beautiful*). Ratings of symmetrical patterns are shown as a dashed line; ratings of asymmetrical patterns are shown as a solid line.

Because of a recording problem, data of one participant were missing. Therefore, we analyzed 78 participants. The ratings were analyzed by a GLMM. Before a detailed explanation of the model and its results, we will briefly summarize the outcome: Participants rated symmetrical patterns significantly higher than asymmetrical ones. In addition, a significant interaction between the patterns’ symmetry and the centered sum of scores of the expertise questionnaire showed that the ratings for asymmetrical patterns significantly increased with higher art expertise (though overall not being higher than the ratings for symmetrical patterns, see Figure 5).

The GLMM will be reported with *p* values based on Satterthwaite approximation. A contrast for the patterns’ symmetry (symmetrical – asymmetrical) and the sum of scores of the expertise questionnaire (as a centered, continuous variable) were included as fixed factors. The interaction between these two factors was included, too. As random factors, we used random by-participants intercepts and slopes for symmetry and random by-pattern intercepts and slopes for the centered sums of scores. Homoscedasticity and normality of model residuals were confirmed by visual inspection of residual plots. There was no correlation of the fixed effects, with *r* = .00.

The GLMM showed a significant effect for symmetrical minus asymmetrical patterns, *b* = 1.41 (95% CI: [1.05, 1.77]), *SE* = 0.18, *t*(121.86) = 7.81, *p* < .001. The ratings for symmetrical patterns were significantly higher compared with the ratings for asymmetrical patterns: The estimated means were 4.24 (95% CI: [3.98, 4.51]) for the symmetrical patterns and 2.83 (95% CI: [2.60, 3.07]) for the asymmetrical patterns. In addition, there was a significant interaction between symmetry and the centered sum of scores, *b* = −0.05, *SE* = 0.02, *t*(78.02) = −2.35, *p* = .022, as shown in Figure 5. The ratings for asymmetrical patterns significantly increased with higher centered sums of scores. More precisely, comparing the slopes of the symmetrical versus the asymmetrical patterns showed that the CI of the slope of the symmetrical patterns, *trend*(77.82) = −0.01, *SE* = 0.02, included zero (95% CI: [−0.04, 0.02]) demonstrating a nonsignificant negative slope. In contrast, the CI of the asymmetrical patterns’ slope, *trend*(78.37) = 0.04, *SE* = 0.01, did not include zero (95% CI: [0.01, 0.06]) showing a significant increase in ratings for asymmetrical patterns with higher centered sum of scores. Table 3 shows the fixed effect results.

**Table 3.** Fixed Effect Results of the Linear Mixed Model for the Explicit Ratings.

	*b*	*SE*	*df*	*t*	*p*
Intercept	3.54	0.09	123.03	39.93	<.001
Difference between symmetrical and asymmetrical patterns	1.41	0.18	121.86	7.81	<.001
Centered sum of scores	0.01	0.01	78.11	1.21	.232
Interaction between centered sum of scores and the difference between symmetrical and asymmetrical patterns	−0.05	0.02	78.02	−2.35	.022

### Implicit Association Test

We analyzed all 79 participants as no one made more than 20% errors. Trials with a wrong answer and trials with a correct RT exceeding 2.5 *SD*s from the mean of the correct RTs per participant and per condition were excluded. Based on these criteria, in total 9.30% of the trials were excluded. To ensure the RTs’ approximately normal distribution, RTs were inverse transformed and multiplied by −1,000 to maintain the direction of effects (x_transformed_ = −1,000/x_original_).

Summarizing the results (to be explained in more detail later), we found faster RTs in the compatible compared with the incompatible block and faster RTs for repetition compared with switch trials. In addition, despite a significant interaction between compatibility and task switching, the difference between the compatible and the incompatible block was significant for repetition and switching trials, and, both in the compatible and the incompatible block, there was a significant difference between switching and repetition trials.

RTs were analyzed by a GLMM with *p* values based on Satterthwaite approximation. Contrasts for compatibility (incompatible – compatible block) and task switching (repetition – switching trials) as well as the sum of scores of the expertise questionnaire (as a centered, continuous variable) were included as fixed factors. The interactions between these factors were included, too. As random factors, we included random by-participants intercepts and slopes for compatibility and task switching and the interaction of both variables, and random by-stimulus intercepts and slopes for compatibility and task switching and, again, the interaction of both variables. The centered sum of scores was not included as a random slope because otherwise the model would not have converged. Homoscedasticity and normality of model residuals were confirmed by visual inspection of residual plots. The correlations of the fixed effects were low (*r* = .00) for the correlation between the centered sum of scores and compatibility and the correlation between the sum of scores and task switching as well as *r* = −.16 for the correlation between compatibility and task switching.

**Figure 6.**
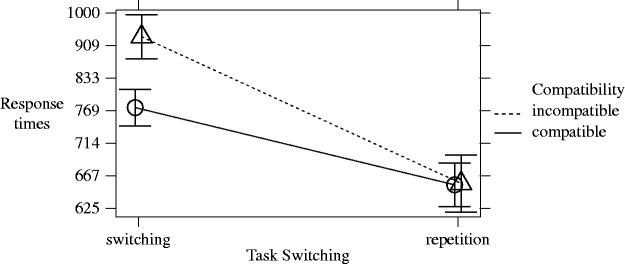
Interaction between task switching and compatibility of the IAT. Response times are back transformed to ms. Trials of the incompatible block are shown as dashed line; trials of the compatible block are shown as solid line.

The GLMM showed a significant effect for the incompatible minus compatible block, *b* = 0.22 (95% CI: [0.18, 0.25]), *SE* = 0.02, *t*(78.82) = 11.60, *p* < .001. RTs were faster in the compatible compared with the incompatible block. The estimated, back transformed means were 715 ms (95% CI: [685 ms, 746 ms]) for the compatible block and 846 ms (95% CI: [806 ms, 893 ms]) for the incompatible block. There was a significant effect for the repetition minus switching trials, *b* = −0.24 (95% CI: [−0.26, −0.22]), *SE* = 0.01, *t*(70.57) = −21.23, *p* < .001, with faster RTs in repetition than switching trials. The estimated, back transformed means were 707 ms (95% CI: [680 ms, 741 ms]) for the repetition trials and 853 ms (95% CI: [813 ms, 893 ms]) for the switching trials. In addition, there was a significant interaction between the difference between incompatible and compatible block and the difference between repetition minus switching trials, *b* = −0.21, *SE* = 0.02, *t*(70.65) = −12.28, *p* < .001. Figure 6 shows this interaction. The task switching conditions led to slower responses in incompatible, *b = *0.3 (95% CI: [0.30, 0.38]), *SE* = 0.02, *t*(78.4) = 20.13, *p* < .001, for the difference between switching and repetition trials, as well as compatible, *b = *0.1 (95% CI: [0.10, 0.15]), *SE* = 0.01, *t*(58.2) = 12.54, *p* < .001, trials. The difference between the compatible and the incompatible block was significant for the repetition, *b* = −0.1 (95% CI: [−0.15, −0.07]), *SE* = 0.02, *t*(79.5) = −6.10, *p* < .001, as well as for the switching, *b* = −0.3 (95% CI: [−0.37, −0.28]), *SE* = 0.02, *t*(83.1) = −14.17, *p* < .001, trials. The back transformed means of all conditions based on compatibility and task switching are shown in Table 4. Table 5 shows the fixed effect results.

**Table 4.** Estimated Means Based on the Linear Mixed Model for Response Times (RTs) of the Implicit Association Test (Back Transformed to ms).

	Mean response time (ms)	95% CI (ms)
Compatible, switching trials	750	[719, 784]
Incompatible, switching trials	990	[935, 1,049]
Compatible, repetition trials	683	[654, 713]
Incompatible, repetition trials	739	[704, 777]

**Table 5.** Fixed Effect Results of the Linear Mixed Model for the Response Times (RTs) of the Implicit Association Test.

	*b*	*SE*	*df*	*t*	*p*
Intercept	−1.29	0.03	92.80	−45.07	<.001
Difference between incompatible and compatible block	0.22	0.02	78.82	11.60	<.001
Centered sum of scores	0.00	0.00	76.99	−0.38	.705
Difference between repetition and switching trials	−0.24	0.01	70.57	−21.23	<.001
Interaction between centered sum of scores and the difference between incompatible and compatible block	0.00	0.00	77.00	0.59	.557
Interaction between the difference between incompatible and compatible block and the difference between repetition and switch trials	−0.21	0.02	70.65	−12.28	<.001
Interaction between centered sum of scores and the difference between repetition and switching trials	0.00	0.00	77.17	−1.79	.078
Interaction between centered sum of scores, the difference between incompatible and compatible block, and the difference between repetition and switching trials	0.00	0.00	76.85	−1.76	.083

#### Error analysis

We analyzed the arcsine-square root transformed error rates by a GLMM identical to the one for the RTs of the IAT as described earlier. Homoscedasticity and normality of model residuals were confirmed by visual inspection. There was no correlation between fixed effects, all *r*s = .00.

Summing up the results of the error rate analysis, they were in accordance with the RT results: The model showed a significant effect for the incompatible minus compatible block, *b* = 0.08 (95% CI: [0.06, 0.10]), *SE* = 0.01, *t*(58.49) = 6.69, *p* < .001. Participants made more errors in the incompatible compared with the compatible block. The estimated means were 0.09 (95% CI: [0.05, 0.13]) for the compatible block and 0.17 (95% CI: [0.13, 0.21]) for the incompatible block. There was a significant effect for the repetition minus switching trials, *b* = −0.06 (95% CI: [−0.08, −0.05]), *SE* = 0.01, *t*(47.43) = −6.21, *p* < .001. The error rate was higher in switching compared with repetition trials. The estimated means were 0.09 (95% CI: [0.06, 0.13]) for the repetition trials and 0.16 (95% CI: [0.12, 0.20]) for the switching trials. In addition, there was a significant interaction between compatibility and task switching, *b* = −0.13, *SE* = 0.02, *t*(77.50) = −6.56, *p* < .001. There was a switching cost in incompatible, *b* = 0.1 (95% CI: [0.09, 0.16]), *SE* = 0.02, *t*(57.10) = 7.88, *p* < .001, but not in compatible, *p* = .900, trials. The difference between the compatible and the incompatible block was not significant for the repetition, *p = *.200, but for the switching, *b* = −0.1 (95% CI: [−0.18, −0.11]), *SE* = 0.02, *t*(66.40) = −7.86, *p* < .001, trials. The means of all conditions based on compatibility and task switching are shown in Table 6. Table 7 shows the fixed effect results.

**Table 6.** Estimated Means Based on the Linear Mixed Model for the Arcsine-Square Root Transformed Error Rates of the Implicit Association Test.

	Mean	95% CI
Compatible, switching trials	0.09	[0.05, 0.12]
Incompatible, switching trials	0.23	[0.18, 0.28]
Compatible, repetition trials	0.09	[0.04, 0.13]
Incompatible, repetition trials	0.10	[0.06, 0.14]

**Table 7.** Fixed Effect Results of the Linear Mixed Model for the Arcsine-Square Root Transformed Error Rates of the Implicit Association Test.

	*b*	*SE*	*df*	*t*	*p*
Intercept	0.13	0.02	44.59	6.85	<.001
Difference between incompatible and compatible block	0.08	0.01	58.49	6.69	<.001
Centered sum of scores	0.00	0.00	76.81	0.54	.589
Difference between repetition and switching trials	−0.06	0.01	47.43	−6.21	<.001
Interaction between centered sum of scores and the difference between incompatible and compatible block	0.00	0.00	82.65	1.40	.165
Interaction between the difference between incompatible and compatible block and the difference between repetition and switching trials	−0.13	0.02	77.50	−6.56	<.001
Interaction between centered sum of scores and the difference between repetition and switching trials	0.00	0.00	158.60	−1.12	.263
Interaction between centered sum of scores, the difference between incompatible and compatible block, and the difference between repetition and switching trials	0.00	0.00	112.90	−1.33	.186

#### Complementary analyses: *D* scores and perceptual fluency

**Figure 7.**
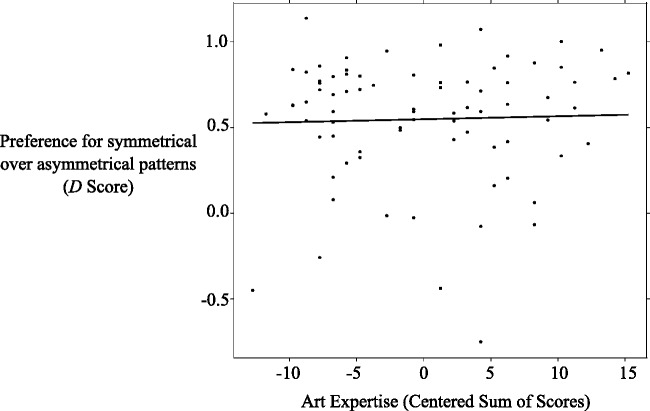
(Nonsignificant) relationship between *D* scores of the IAT and the centered sum of scores of the art-expertise questionnaire. A higher centered sum of scores signifies higher art expertise. Positive *D* scores indicate faster RTs in the compatible compared with the incompatible block.

*D* scores are an often used standardized measure of the IAT effect (cf. Makin et al., 2012). They represent the difference between incompatible and compatible RTs divided by the pooled *SD* per participant. Analyzing *D* scores (*M* = 0.55, 95% CI: [0.47, 0.63]) revealed that they are significantly larger than zero, *t*(78) = 13.55, *p* < .001, *d* = 1.52. This indicated that RTs in the compatible blocks were, overall, shorter compared with the incompatible blocks. Interestingly, 8 out of the 79 participants showed a negative *D* score indicating faster RTs in the incompatible compared with the compatible block. However, *D* scores did not correlate with the centered sum of scores of the art-expertise questionnaire, *r* = .03, *r*^2 ^= .001, *p* = .761 (see Figure 7).

Similarly to Makin et al. (2012), we analyzed the role of fluency (cf. Reber, Schwarz, et al., 2004) for implicit preferences. Looking at the RTs in the training blocks for patterns (Blocks 2 and 4; see Table 2), the mean RT for symmetrical patterns was faster (989 ms) than for asymmetrical patterns (1,049 ms), although they did not differ significantly, *t*(155.86) = −1.36, *p* = .176, *d* = −0.22. A one-sample *t* test of the difference between asymmetrical and symmetrical patterns (*M_difference_* = 61 ms, 95% CI: [16, 105]) revealed a significant result, *t*(78) = 2.73, *p* = .008, *d* = 0.31. Out of the 79 participants, 27 showed a faster mean RT with asymmetrical compared with symmetrical patterns, but the difference between symmetrical and asymmetrical patterns in the training blocks did not correlate with the centered sum of scores, *r* = −.11, *r*^2 ^= −.01, *p* = .356. In addition, we calculated the correlation between *D* scores and the difference between asymmetrical and symmetrical patterns in the training blocks (cf. Makin et al., 2012). If symmetry preference is in line with a more fluent processing of symmetrical compared with asymmetrical patterns, we could possibly expect a positive correlation between *D* scores and the RT advantage for symmetrical over asymmetrical patterns (cf. Makin et al., 2012). The correlation was not significant with *r* = −.03, *r*^2 ^= −.001, *p* = .818. This was the same when only analyzing participants with a slower RT in asymmetrical compared with symmetrical patterns in the training blocks.

## Discussion

The present study investigated whether preference for symmetry is dependent on art expertise. It revealed that there is a strong implicit preference for symmetry independent of art expertise. In contrast, explicit preferences change with expertise. Specifically art expertise is associated with a slight increase in preference for asymmetrical patterns (although even experts still like symmetry more than asymmetry). In the following, we will discuss these results in a broader scientific context.

It is long known that, perceptually, visual symmetry stands out as a good Gestalt feature (Köhler, 1929). Processing of mirror-symmetrical stimuli is faster than processing of comparable asymmetrical stimuli (Barlow & Reeves, 1979; Julesz, 1971; for a review, see Wagemans, 1995), helping to group and segregate the visual input into perceptual objects and background (Machilsen, Pauwels, & Wagemans, 2009). Together with the felt ease or fluency of symmetry processing, these factors could be responsible for human symmetry preferences in aesthetic appreciation (Reber, Schwarz, et al., 2004).

However, as some complexity is also appreciated (McManus, 2005) but negatively correlated with symmetry (Gartus & Leder, 2017; Nadal, Munar, Marty, & Cela-Conde, 2010), and as humans generally differ in their appreciation of visual objects, it stands to reason if symmetrical patterns are generally preferred compared with asymmetrical patterns. The aim of our study was therefore to test whether visual symmetry is generally preferred over visual asymmetry (cf. Makin et al., 2012). To that end, we carefully discriminated between explicit liking ratings and implicit preference measures because fast and automatic preferences that are implicitly measured might show a different picture than the more reflected explicit judgments. In fact, this difference in measures also had an effect on the degree of symmetry preferences in the present study. While participants with higher art expertise and lower art expertise showed similar preferences in their implicit ratings, art experts showed less of a symmetry preference in their explicit ratings only.

In detail, participants had different levels of art expertise as revealed by an art-expertise questionnaire. Symmetry preference was measured explicitly by a rating scale and implicitly by a variant of the IAT (Greenwald et al., 1998). The explicit rating scale revealed that participants preferred symmetrical over asymmetrical patterns independent of their sum of scores of the art-expertise questionnaire. However, the explicit rating scale also showed an interaction between art expertise and symmetry preference: A higher art-expertise score was associated with higher ratings for asymmetrical patterns. (Keep in mind however, that overall, also art experts did not rate asymmetrical patterns higher than symmetrical ones: There was no significant change dependent on the sum of scores in ratings for symmetrical patterns). We, therefore, conclude that higher art expertise goes hand in hand with higher declared beauty ratings of asymmetry.

Interestingly, this interaction was not found in the IAT: Indicative of a symmetry preference, we found shorter RTs in the compatible block compared with the incompatible block. Again, in the complementary analyses, we found no interaction between the *D* score (representing the standardized RT difference between the incompatible and the compatible block) and art expertise. This means that symmetrical patterns were generally linked with positive valence and asymmetrical patterns with negative valence. This was the case to roughly the same extent regardless of measured art expertise.

In addition, we made sure that our IAT task measure symmetry preferences in an implicit way rather than only by means of a deliberate recoding of the picture- versus word-discrimination responses into one task in the compatible blocks and into two separate tasks in the incompatible blocks. Admittedly, in the IAT task, we found a significant interaction between task switching and compatibility: The switching cost (the delayed RT where the stimulus category changed, for instance, when a word followed a pattern as compared with where the stimulus category repeated) was higher in incompatible than in compatible blocks and the difference between the compatible and the incompatible block was larger in switching trials than repetition trials. This is evidence of a deliberate recoding of the tasks. However, nevertheless, in the RTs, there was a switching cost in compatible blocks, too, and the RT difference between the compatible and the incompatible block also existed for repetition trials (where the stimulus category did not change). Also, these RT effects were not due to a speed-accuracy trade-off as the error rates did not show evidence of reversed effects. This means that our IAT effect (the difference in RTs between the incompatible and the compatible block) does not only stem from the switching trials and, therefore, is not solely based on deliberate task recoding (cf. Mierke & Klauer, 2001). This in turn means that our interpretation of the IAT effect as a more implicit measure is valid.

What might be the possible reasons for a higher explicit beauty rating for asymmetrical patterns by participants with higher art expertise? We propose that a symmetry preference is based on evolutionary adaptation, similarly for almost everybody (cf. Cárdenas & Harris, 2006): Symmetry might symbolize “good” genes or, for instance, in faces, the quality of a possible mating partner (Møller, 1992; but see Makin, Bertamini, Jones, Holmes, & Zanker, 2016; Van Dongen, 2011). As a valid indicator of health, a symmetry preference would thus lead to healthier offspring and finally to a higher reproductive success and a stronger presence of symmetry preferences in the phenotype of the population (Grammer, Fink, Møller, & Thornhill, 2003). In addition to evolutionary shaped preferences, fluently (or easily) processed (cf. Reber, Schwarz et al., 2004), prototypical (cf. Martindale, Moore, & Borkum, 1990), or familiar objects (cf. Zajonc, 1968) are preferred for the lower effort needed to process them (cf. Winkielman, Halberstadt, Fazendeiro, & Catty, 2006; for a discussion of this topic, see also Leder et al., 2004; Lindell & Mueller, 2011; Silvia, 2006). However, liking and preferences based on fluency, prototypicality, or familiarity have in common that they could vary inter- or even intraindividually (the latter, across time). Lindell and Mueller (2011) suggested that “the importance of symmetry on judgments of aesthetic beauty decreases as artistic training increases, presumably reflecting the fact that artistic training enables more fluent processing of even complex works” (p. 459). In addition, art experts might be more familiar with asymmetrical patterns, these patterns might be more prototypical to them (cf. McManus, 2005), and, therefore, they might be able to process them more fluently and like them more.

However, this does not fully align with the absent interaction between art expertise and preference when measured implicitly (cf. Winkielman et al., 2006). In addition, we did not find any evidence for a more fluent processing of one type of patterns as shown in the complementary analyses of the IAT. This analysis was carried out similarly to Makin et al. (2012). Interestingly, when these authors analyzed their experiments separately, they also did not find a more fluent processing of symmetrical patterns compared with asymmetrical (“*random*”) patterns (see results of Experiment 1 in Table 2 of Makin et al., 2012).

Thus, an alternative account is more plausible. Art experts might, similarly to nonexperts, prefer symmetrical over asymmetrical patterns. This preference could even be automatic, reflected in implicit measures, in our case the IAT. Yet, the explicit ratings might have elicited a more deliberate process of “*cognitive mastering*” (Leder et al., 2004). As outlined in the Introduction, asymmetrical depictions play an important role in the history of arts, and especially art experts might therefore appreciate slight deviations from symmetry (McManus, 2005). Lindell and Mueller (2011) even raised the question whether art experts might seek to set themselves apart from the hoi polloi: They “seek to distinguish their penchants from those of the masses” (p. 466). We would therefore reason that the higher ratings for asymmetrical patterns by art experts are based on an explicit consideration complementing the implicit liking for symmetrical patterns (cf. Leder et al., 2004; Lindell & Mueller, 2011). Related to this possibility, art experts sometimes show differences between their initial impressions and their explicit reflections based on cognitive revisions of these first impressions (Leder et al., 2004, 2014). And this might also have affected their explicit judgments in the present study. This account seems even more plausible when recalling the fact that there was no time limit on answers in the explicit rating task, but quick and correct responses were required in the IAT task. If a symmetry preference is a quick response and an explicit rating is based on subsequent revisions of the quick first impressions in the context of higher cognitive processes (Leder et al., 2004), in the IAT task setting, the experts would simply not have had the time for their revisions.

In addition, other possible influences of the experimental setting have to be kept in mind: First, we used abstract patterns and not artworks. Because artworks are almost always asymmetrical depictions (cf. McManus, 2005), they might even provoke a higher valence of asymmetrical over symmetrical patterns. Furthermore, we presented the patterns in a controlled experimental setting—on a monitor in our laboratory—which is not the classical setting for the appreciation of artworks, as for instance, would be a museum. Context is an important factor in the appreciation of artwork (Brieber, Nadal, & Leder, 2015; Locher, Smith, & Smith, 2001). For example, context helps to recognize an object as art (Leder et al., 2004). In our study, participants were not asked to evaluate the patterns as being objects of art. We wanted to measure symmetry preferences for art experts and nonexperts as it is usually done in studies on symmetry preference under conditions allowing a high degree of control over the extent of symmetry in an image but without an “*artistic context*”. It is possible that by evoking (or *priming* of) an artistic context in a museum, art experts could even be prompted to implicitly evaluate asymmetry higher or that even nonexperts would then start to show higher explicit asymmetry preferences.

Furthermore, only in the IAT task, patterns were presented repeatedly, and such repetitions have the potential to increase liking. Yet, this should have made all of our patterns more likeable, not just symmetrical ones—a fact that is at odds with our finding of a higher preference for symmetrical over asymmetrical patterns in the IAT.

However, we are well aware of the fact that, although we controlled for the complexity (cf. Silvia, 2006) and some other varying features of the patterns by means of the GLMM, we did not take into account the visual balance of the stimuli. Visual balance specifies whether the *weight* of an image is evenly distributed. Whereas a symmetrical pattern is usually balanced, an asymmetrical pattern can be balanced if the visual weight is evenly distributed although the shapes are not identically mirrored (cf. Lok, Feiner, & Ngai, 2004). Thus, balance seems to be generally confounded with symmetry and future research could try to disentangle influences of symmetry and balance.

Lastly, it is an ongoing debate how much the preferences measured in the current study represent aesthetic experiences in real-world settings, like art exhibitions (Makin, 2017). Some authors claim that strong emotional feelings accompany such real-world experiences (Pelowski, 2015), and it is doubtful if such experiences are ever elicited in the laboratory. Thus, it remains a major task to systematically compare outcomes of laboratory studies with real-world experiences to finally decide how much of the current findings are valid (Pelowski, Forster, Tinio, Scholl, & Leder, 2017; Pelowski, Markey, Forster, Gerger, & Leder, 2017).

To conclude, our study revealed that the generality of the symmetry preference is not as strongly present when art experts explicitly report their preferences. This is in line with an interactionist perspective on art evaluation that takes into account the evaluated object and the person looking at it (cf. Leder et al., 2004; Reber, Schwarz, et al., 2004). General preferences (e.g., shaped through evolution) and personal history jointly shape human aesthetic preferences for symmetry or asymmetry.

## Appendix A

### Art-Expertise Questionnaire

In the following, we present the two parts of the computer version of the art-expertise questionnaire that we analyzed for our study. The English translation is written in italics. Participants had to answer all questions. The second part consisted of the following works of art: Salvador Dalí—Soft Construction with Boiled Beans (Premonition of Civil War), Andy Warhol—Banana (from the Velvet Underground’s debut album cover), Keith Haring—Best Buddies, Marcel Duchamp—Fountain, Claude Monet—The Water-Lily Pond, Caspar David Friedrich—Wanderer Above the Sea Fog, Leonardo da Vinci—Lady with an Ermine, and Carl Spitzweg—The Bookworm. Participants were not given these names but were presented the art works as printed images.

### First Part

In diesem Abschnitt finden Sie zehn verschiedene Fragen zum Thema Kunst.

Bitte wählen Sie immer eine Antwort aus den vier möglichen aus.

*In this section, you will find 10 questions regarding art. Please choose one out of four possible answers*.

Welcher Maler verlor durch einen gewaltsamen Zwischenfall ein Ohr?

*Which painter lost his ear in a violent incident?*

- Van Gogh
- Munch
- Renoir
- Dali

Wer bemalte vor allem die Sixtinische Kapelle im Vatikan?

*Who painted mainly the Sistine Chapel at the Vatican?*

- Michelangelo
- Da Vinci
- Van Gogh
- Raffael

Wer malte die Mona Lisa?

*Who painted the Mona Lisa?*

- Picasso
- Monet
- Da Vinci
- Michelangelo

Bernd und Hilla Becher sind bekannt für ihre …

*Bernd and Hilla Becher are known for their …*

- Schw.-weiß-Fotografien *(black-and-white photography)*
- Stahlskulpturen *(steel sculptures)*
- Aquarelle *(aquarelles)*
- Tanzperformances *(dance performances)*

Wer malte das Gemälde “Nachtwache”?

*Who painted the “Night Watch”?*

- Rembrandt
- Van Gogh
- Da Vinci
- Hohlbein

Wer wurde durch seine Plakatmalerei im 19. Jahrhundert bekannt?

*Who became famous for his poster paintings in the 19th century?*

- Toulouse-Lautrec
- Gaudi
- Dalí
- Berthon

Welche Stadt gilt seit über 50 Jahren als die größte Kunstmetropole?

*Which city has been known as the biggest art metropolis for more than 50 years?*

- New York
- Barcelona
- Amsterdam
- London

Wo wurde die Künstlergruppe “die Brücke” gegründet?

*Where was the artist group “The Bridge” founded?*

- Dresden
- München
- Hamburg
- Berlin

Die Schaffensphase von Picasso zwischen 1901 und 1904 nennt man die …

*Picasso’s creative period between 1901 and 1904 is called the …*

- blaue Periode *(blue period)*
- rote Periode *(red period)*
- grüne Phase *(green period)*
- gelbe Phase *(yellow period)*

Wer dehnte in dem Werk “24 hour psycho” einen Hitchcock-Film auf 24 Stunden aus?

*Who extended a Hitchcock film to 24 hours in their work “24 hour psycho”?*

- Douglas Gordon
- Pipilotti Rist
- Nam June Paik
- Joseph Beuys

### Second Part

Anschließend sehen Sie sich bitte der Reihe nach die Abbildungen in der beigelegten Mappe an. Geben Sie zunächst an, ob Ihnen das jeweilige abgebildete Kunstwerk bekannt ist: “Ja” bzw. “Nein.” Falls möglich geben Sie bitte auch an, von welchem Künstler/welcher Künstlerin das jeweilige Kunstwerk stammt und mit welcher Stilrichtung das Kunstwerk hauptsächlich in Verbindung gebracht wird. Falls nicht möglich, markieren Sie bitte die entsprechende Zelle ebenfalls mit einem “Nein.”

*Subsequently, please inspect the images in the attached folder, one after the other. Then indicate if you know the shown artwork: “Yes” or “No.” If possible, please also indicate the painter of the artwork and the style with which it is associated primarily. If not possible, please enter “No” into the corresponding cell.*

Beispiel: *(Example:)*

Das Bild hat man schon mal gesehen: “Ja”

*You have seen the image before: “Yes”*

Künstler ist einem bekannt: “Name des Künstlers”

*You know the artist: “Name of the artist”*

Die Kunstrichtung ist jedoch unbekannt: “Nein”

*However, you do not know the style: “No”*

Bild 1 – 8: *(Image 1–8:)*

Ist das Bild bekannt? (“Ja” bzw. “Nein”)

*Do you know the image (“Yes” or “No”)*

KünstlerIn: *(artist:)*

Kunstrichtung/Epoche/Stil: *(art form/epoch/style:)*

## References

[bibr1-2041669518761464] Allport A. Styles E. A. Hsieh S. (1994) Shifting intentional set: Exploring the dynamic control of tasks. In: Umiltà C. Moscovitch M. (eds) Conscious and nonconscious information processing: Attention and performance XV, Cambridge, MA: MIT Press, pp. 421–452.

[bibr64-2041669518761464] Barron, F. (1952). Artistic perception as a factor in personality style. *Journal of Psychology*, *33*, 199–203.

[bibr2-2041669518761464] Barlow H. B. Reeves B. C. (1979) The versatility and absolute efficiency of detecting mirror symmetry in random dot displays. Vision Research 19: 783–793. doi:10.1016/0042-6989(79)90154-8. 483597 10.1016/0042-6989(79)90154-8

[bibr3-2041669518761464] Brieber D. Nadal M. Leder H. (2015) In the white cube: Museum context enhances the valuation and memory of art. Acta Psychologica 154: 36–42. doi:10.1016/j.actpsy.2014.11.004. 25481660 10.1016/j.actpsy.2014.11.004

[bibr4-2041669518761464] Cárdenas R. A. Harris L. J. (2006) Symmetrical decorations enhance the attractiveness of faces and abstract designs. Evolution and Human Behavior 27: 1–18. doi:10.1016/j.evolhumbehav.2005.05.002.

[bibr5-2041669518761464] Darvas G. (2003) Perspective as a symmetry transformation. Nexus Network Journal 5: 9–21. doi:10.1007/s00004-002-0002-8.

[bibr6-2041669518761464] Fox J. (2003) Effect displays in R for generalised linear models. Journal of Statistical Software 8: 1–27. doi:10.18637/jss.v008.i15.

[bibr7-2041669518761464] Gartus A. Leder H. (2013) The small step toward asymmetry: Aesthetic judgment of broken symmetries. i-Perception 4: 352–355. doi:10.1068/i0588sas. 24349695 10.1068/i0588sas PMC3859553

[bibr8-2041669518761464] Gartus A. Leder H. (2017) Predicting perceived visual complexity of abstract patterns using computational measures: The influence of mirror symmetry on complexity perception. PLoS One 12: 1–29. doi:10.1371/journal.pone.0185276. 10.1371/journal.pone.0185276 PMC5669424 29099832

[bibr9-2041669518761464] Grammer K. Fink B. Møller A. P. Thornhill R. (2003) Darwinian aesthetics: Sexual selection and the biology of beauty. Biological Reviews 78: 385– 407. doi:10.1017/S1464793102006085. 14558590 10.1017/s1464793102006085

[bibr10-2041669518761464] Greenwald A. G. McGhee D. E. Schwartz J. L. K. (1998) Measuring individual differences in implicit cognition: The Implicit Association Test. Journal of Personality and Social Psychology 74: 1464–1480. doi:10.1037/0022-3514.74.6.1464. 9654756 10.1037//0022-3514.74.6.1464

[bibr11-2041669518761464] Halberstadt J. Rhodes G. (2003) It’s not just average faces that are attractive: Computer-manipulated averageness makes birds, fish, and automobiles attractive. Psychonomic Bulletin & Review 10: 149–156. doi:10.3758/BF03196479. 12747502 10.3758/bf03196479

[bibr12-2041669518761464] Hubbell M. B. (1940) Configurational properties considered “good” by naïve subjects. American Journal of Psychology 53: 46–69. doi:10.2307/1415960.

[bibr13-2041669518761464] Jacobsen T. Höfel L. (2001) Aesthetics electrified: An analysis of descriptive symmetry and evaluative aesthetic judgment processes using event-related brain potentials. Empirical Studies of the Arts 19: 177–190. doi:10.2190/P7W1-5F1F-NJK9-X05B.

[bibr14-2041669518761464] Jacobsen T. Höfel L. (2002) Aesthetic judgments of novel graphic patterns: Analyses of individual judgments. Perceptual and Motor Skills 95: 755–766. doi:10.2466/PMS.95.7.755-766. 12509172 10.2466/pms.2002.95.3.755

[bibr15-2041669518761464] Jersild, A. T. (1927). *Mental set and shift. Archives of psychology, Whole No. 89*.

[bibr16-2041669518761464] Julesz B. (1971) Foundations of cyclopean perception, Chicago, IL: University of Chicago Press.

[bibr17-2041669518761464] Köhler W. (1929) Gestalt psychology, New York, NY: Liveright.

[bibr18-2041669518761464] Kray J. Lindenberger U. (2000) Adult age differences in task switching. Psychology and Aging 15: 126–147. doi:10.1037/0882-7974.15.1.126. 10755295 10.1037//0882-7974.15.1.126

[bibr19-2041669518761464] Kuznetsova, A., Brockhoff, P. B., & Christensen, R. H. B. (2016). lmerTest: Tests in Linear Mixed Effects Models (Version 2.0.33) [Software, R package]. Retrieved from https://CRAN.R-project.org/package=lmerTest.

[bibr20-2041669518761464] Leder H. Belke B. Oeberst A. Augustin D. (2004) A model of aesthetic appreciation and aesthetic judgments. British Journal of Psychology 95: 489–508. doi:10.1348/0007126042369811. 15527534 10.1348/0007126042369811

[bibr21-2041669518761464] Leder H. Gerger G. Brieber D. Schwarz N. (2014) What makes an art expert? Emotion and evaluation in art appreciation. Cognition and Emotion 28: 1137–1147. doi:10.1080/02699931.2013.870132. 24383619 10.1080/02699931.2013.870132

[bibr22-2041669518761464] Lenth R. V. (2016) Least-squares means: The R package lsmeans. Journal of Statistical Software 69: 1–33. doi:10.18637/jss.v069.i01.

[bibr23-2041669518761464] Lidwell W. Holden K. Butler J. (2010) Universal principles of design, Beverly, MA: Rockport Publishers.

[bibr24-2041669518761464] Lindell A. K. Mueller J. (2011) Can science account for taste? Psychological insights into art appreciation. Journal of Cognitive Psychology 23: 453–475. doi:10.1080/20445911.2011.539556.

[bibr25-2041669518761464] Locher P. J. Smith J. K. Smith L. F. (2001) The influence of presentation format and viewer training in the visual arts on the perception of pictorial and aesthetic qualities of paintings. Perception 30: 449–465. doi:10.1068/p3008. 11383192 10.1068/p3008

[bibr26-2041669518761464] Lok S. Feiner S. Ngai G. (2004) Evaluation of visual balance for automated layout. *In:* Proceedings of the 9th International Conference on Intelligent User Interfaces, Madeira, Portugal: Association for Computing Machinery, pp. 101–108.

[bibr27-2041669518761464] Machilsen B. Pauwels M. Wagemans J. (2009) The role of vertical mirror symmetry in visual shape detection. Journal of Vision 9((12): 1–11. doi:10.1167/9.12.11. 10.1167/9.12.11 20053102

[bibr28-2041669518761464] Makin A. D. J. (2017) The gap between aesthetic science and aesthetic experience. Journal of Consciousness Studies 24: 184–213.

[bibr29-2041669518761464] Makin A. D. J. Bertamini M. Jones A. Holmes T. Zanker J. M. (2016) A gaze-driven evolutionary algorithm to study aesthetic evaluation of visual symmetry. i-Perception 7: 1–18. doi:10.1177/2041669516637432. 10.1177/2041669516637432 PMC4934674 27433324

[bibr30-2041669518761464] Makin A. D. J. Pecchinenda A. Bertamini M. (2012) Implicit affective evaluation of visual symmetry. Emotion 12: 1021–1030. doi:10.1037/a0026924. 22251051 10.1037/a0026924

[bibr31-2041669518761464] Martindale C. Moore K. Borkum J. (1990) Aesthetic preference: Anomalous findings for Berlyne’s psychobiological theory. American Journal of Psychology 103: 53–80. doi:10.2307/1423259.

[bibr32-2041669518761464] McManus I. C. (2005) Symmetry and asymmetry in aesthetics and the arts. European Review 13(Suppl. 2): 157–180. doi:10.1017/S1062798705000736.

[bibr33-2041669518761464] McWhinnie H. J. (1968) A review of research on aesthetic measure. Acta Psychologica 28: 363–375. doi:10.1016/0001-6918(68)90025-5.

[bibr34-2041669518761464] Mierke J. Klauer K. C. (2001) Implicit association measurement with the IAT: Evidence for effects of executive control processes. Experimental Psychology 48: 107–122. doi:10.1026//0949-3946.48.2.107. 10.1026//0949-3946.48.2.107 11392979

[bibr35-2041669518761464] Mierke J. Klauer K. C. (2003) Method-specific variance in the Implicit Association Test. Journal of Personality and Social Psychology 85: 1180–1192. doi:10.1037/0022-3514.85.6.1180. 14674823 10.1037/0022-3514.85.6.1180

[bibr36-2041669518761464] Møller A. P. (1992) Female swallow preference for symmetrical male sexual ornaments. Nature 357: 238–240. doi:10.1038/357238a0. 1589021 10.1038/357238a0

[bibr37-2041669518761464] Nadal M. Munar E. Marty G. Cela-Conde C. J. (2010) Visual complexity and beauty appreciation: Explaining the divergence of results. Empirical Studies of the Arts 28: 173–191. doi:10.2190/EM.28.2.d.

[bibr38-2041669518761464] Nosek B. A. Greenwald A. G. Banaji M. R. (2007) The Implicit Association Test at age 7: A methodological and conceptual review. In: Bargh J. A. (ed.) Automatic processes in social thinking and behavior, Hove, England: Psychology Press, pp. 265–292.

[bibr39-2041669518761464] Pecchinenda A. Bertamini M. Makin A. D. J. Ruta N. (2014) The pleasantness of visual symmetry: Always, never or sometimes. PLoS One 9: 1–10. doi:10.1371/journal.pone.0092685. 10.1371/journal.pone.0092685 PMC3962427 24658112

[bibr40-2041669518761464] Pelowski M. (2015) Tears and transformation: Feeling like crying as an indicator of insightful or “aesthetic” experience with art. Frontiers in Psychology 6: 1–23. doi:10.3389/fpsyg.2015.01006. 26257671 10.3389/fpsyg.2015.01006 PMC4511828

[bibr41-2041669518761464] Pelowski M. Forster M. Tinio P. P. L. Scholl M. Leder H. (2017) Beyond the lab: An examination of key factors influencing interaction with ‘real’ and museum-based art. Psychology of Aesthetics, Creativity, and the Arts 11: 245–264. doi:10.1037/aca0000141.

[bibr42-2041669518761464] Pelowski M. Markey P. S. Forster M. Gerger G. Leder H. (2017) Move me, astonish me … delight my eyes and brain: The Vienna Integrated Model of top-down and bottom-up processes in Art Perception (VIMAP) and corresponding affective, evaluative, and neurophysiological correlates. Physics of Life Reviews 21: 80–125. doi:10.1016/j.plrev.2017.02.003. 28347673 10.1016/j.plrev.2017.02.003

[bibr43-2041669518761464] Pfister, R., & Janczyk, M. (2015). schoRsch: Tools for analyzing factorial experiments (Version 1.2) [Software, R package]. Retrieved from https://CRAN.R-project.org/package=schoRsch.

[bibr44-2041669518761464] R Core Team. (2016). R: A language and environment for statistical computing [Computer software]. Vienna, Austria: R Foundation for Statistical Computing.

[bibr45-2041669518761464] Reber R. Schwarz N. Winkielman P. (2004) Processing fluency and aesthetic pleasure: Is beauty in the perceiver’s processing experience? Personality and Social Psychology Review 8: 364–382. doi:10.1207/s15327957pspr0804_3. 15582859 10.1207/s15327957pspr0804_3

[bibr46-2041669518761464] Reber R. Wurtz P. Zimmermann T. D. (2004) Exploring “fringe” consciousness: The subjective experience of perceptual fluency and its objective bases. Consciousness and Cognition 13: 47–60. doi:10.1016/S1053-8100(03)00049-7. 14990240 10.1016/S1053-8100(03)00049-7

[bibr47-2041669518761464] Rogers R. D. Monsell S. (1995) Costs of a predictable switch between simple cognitive tasks. Journal of Experimental Psychology: General 124: 207–231. doi:10.1037/0096-3445.124.2.207.

[bibr48-2041669518761464] Silvia P. J. (2006) Artistic training and interest in visual art: Applying the appraisal model of aesthetic emotions. Empirical Studies of the Arts 24: 139–161. doi:10.2190/DX8K-6WEA-6WPA-FM84.

[bibr49-2041669518761464] SR Research. (2004). SR Research Experiment Builder [Computer software]. Mississauga, Canada: SR Research Ltd.

[bibr50-2041669518761464] Swaddle J. P. Cuthill I. C. (1994) Preference for symmetric males by female zebra finches. Nature 367: 165–166. doi:10.1038/367165a0.

[bibr51-2041669518761464] Tinio P. P. L. Leder H. (2009) Just how stable are stable aesthetic features? Symmetry, complexity, and the jaws of massive familiarization. Acta Psychologica 130: 241–250. doi:10.1016/j.actpsy.2009.01.001. 19217589 10.1016/j.actpsy.2009.01.001

[bibr52-2041669518761464] Treder M. S. (2010) Behind the looking-glass: A review on human symmetry perception. Symmetry 2: 1510–1543. doi:10.3390/sym2031510.

[bibr53-2041669518761464] Van Dongen S. (2011) Associations between asymmetry and human attractiveness: Possible direct effects of asymmetry and signatures of publication bias. Annals of Human Biology 38: 317–323. doi:10.3109/03014460.2010.544676. 21271817 10.3109/03014460.2010.544676

[bibr54-2041669518761464] Venables W. N. Ripley B. D. (2002) Modern applied statistics with S, New York, NY: Springer.

[bibr55-2041669518761464] Võ M. L.-H. Conrad M. Kuchinke L. Urton K. Hofmann M. J. Jacobs A. M. (2009) The Berlin Affective Word List Reloaded (BAWL – R). Behavior Research Methods 41: 534–538. doi:10.3758/BRM.41.2.534. 19363195 10.3758/BRM.41.2.534

[bibr56-2041669518761464] Võ M. L.-H. Jacobs A. M. Conrad M. (2006) Cross-validating the Berlin Affective Word List. Behavior Research Methods 38: 606–609. doi:10.3758/BF03193892. 17393831 10.3758/bf03193892

[bibr57-2041669518761464] Wagemans J. (1995) Detection of visual symmetries. Spatial Vision 9: 9–32. doi:10.1163/156856895X00098. 7626549 10.1163/156856895x00098

[bibr58-2041669518761464] Washburn D. K. Crowe D. W. (1988) Symmetries of culture: Theory and practice of plane pattern analysis, Seattle, WA: University of Washington Press.

[bibr59-2041669518761464] Weichselbaum, H., Leder, H., & Ansorge, U. (2017). *WeichselbaumLederAnsorge2017* [Data files]. Retrieved from https://doi.org/10.6084/m9.figshare.5505340.

[bibr60-2041669518761464] Weyl H. (1952) Symmetry, Princeton, NJ: University Press.

[bibr61-2041669518761464] Winkielman P. Halberstadt J. Fazendeiro T. Catty S. (2006) Prototypes are attractive because they are easy on the mind. Psychological Science 17: 799–806. doi:10.1111/j.1467-9280.2006.01785.x. 16984298 10.1111/j.1467-9280.2006.01785.x

[bibr62-2041669518761464] Wright D. Bertamini M. (2015) Aesthetic judgements of abstract dynamic configurations. Art & Perception 3: 283–301. doi:10.1163/22134913-00002037.

[bibr63-2041669518761464] Zajonc R. B. (1968) Attitudinal effects of mere exposure. Journal of Personality and Social Psychology 9: 1–27. doi:10.1037/h0025848.5667435

